# Comparative Evaluation of Sealing Ability, Water Absorption, and Solubility of Three Temporary Restorative Materials: An *in vitro* Study

**DOI:** 10.5005/jp-journals-10005-1423

**Published:** 2017-06-01

**Authors:** AR Prabhakar, N Shantha Rani, Saraswathi V Naik

**Affiliations:** 1 Professor and Head, Department of Pedodontics and Preventive Dentistry, Bapuji Dental College & Hospital, Davangere, Karnataka, India; 2 Postgraduate Student, Department of Pedodontics and Preventive Dentistry, Bapuji Dental College & Hospital, Davangere, Karnataka, India; 3 Reader, Department of Pedodontics and Preventive Dentistry, Bapuji Dental College & Hospital, Davangere, Karnataka, India

**Keywords:** Cavit G, GC Caviton, Intermediate restorative material, Sealing ability, Solubility, Temporary restoration, Water absorption.

## Abstract

**Background:**

The quality of the coronal seal of root canal filling material is important for periapical health. Absorption of water or saliva by the temporary restorative materials leads to dimensional changes, loss of retention, staining and breaking in margin contours. Hence this study was carried out to evaluate and compare the sealing properties, water absorption and solubility of IRM (intermediate restorative material), Cavit G and GC Caviton.

**Study design:**

Experimental, in vitro intergroup randomized control trial.

**Material and methods:**

36 non carious premolars were randomly selected assigned to three groups, 12 teeth in each. Standard endodontic access cavities of approximately 4x4mm wide were prepared followed by the root canal obturation with Gutta-percha and restoration with experimental materials. For microleakage testing dye penetration method was used with 2% methylene blue dye. Followed by evaluation and scoring under stereomicroscope at 40x magnification.

Disc shaped 12 specimens for each group were prepared for each material, stored in desiccator at 37° C, weighed daily to verify mass stabilization (dry mass,m1). Thereafter, the specimens were stored in distilled water at 37°C for 7days to obtain the mass after saturation with water (m2). The specimens were placed in the desiccators again, at 37° C, and reweighed until a constant dry mass is obtained (m3). Water absorption (WS) and solubility (SL) was determined by using the formulas, WS = m3 - m2/V and SL= ml - m3/ V.

**Results:**

GC Caviton showed least microleakage and least water absorption followed by IRM and Cavit G, the differences were statistically highly significant ( p < 0.001) and there was no statistical difference found in all the groups with respect to solubility.

**Conclusions:**

GC Caviton is best and suitable temporary restorative material in endodontic interappointments followed by IRM and Cavit G

**How to cite this article:**

Prabhakar AR, Rani NS, Naik SV. Comparative Evaluation of Sealing Ability, Water Absorption, and Solubility of Three Temporary Restorative Materials: An *in vitro* Study. Int J Clin Pediatr Dent 2017;10(2):136-141.

## INTRODUCTION

Bacterial infection is the most common cause of pulpal and periradicular disease. Also, preventing the entrance of bacteria in all phases of endodontic therapy is a basic principle for its success.^1^ In many situations, the treatment cannot be completed in a single session, which might require multiple appointments for which dressing with antibacterial medicaments with effective temporary coronal sealing for different periods of time becomes mandatory.^2^ Thus, a provisional restorative material plays a pivotal role in sealing a root canal and keeping the root canals sterile, thus preventing contamination of food debris, oral fluids, and microbes, which can result in postoperative failure. In addition, they also prevent the escape of medicaments which were placed in the pulp chamber and root canal system.^3^

Temporary filling materials can degrade when exposed to saliva in the mouth, and the resulting gap between the tooth and the restoration predisposes the tooth to caries and periodontal disease. Absorption of water or saliva by these temporary restorative materials leads to dimensional changes, loss of retention, staining, and breaking in margin contours.^4^ Also, lack of satisfactory temporary restorations during endodontic therapy is stated as second contributing factor in continuing pain after commencement of treatment. Coronal microleakage can considerably affect the prognosis of endodontic treatment.^5^

Sealing and low water absorption (WS) and solubility (SL) are desired features of an effective temporary sealing material. Intermediate restorative material (IRM) is a commonly used temporary restorative material, composed of zinc oxide eugenol reinforced with polymethyl methacrylate and has better sealing ability. Cavit G is a premixed temporary restorative material that contains zinc oxide, calcium sulfate, glycol acetate, polyvinyl acetate resins, polyvinyl chloride acetate, triethanolamine, and pigments. It has a high coefficient of linear expansion resulting from water sorption, thus providing good seal.^5^

GC Caviton is a newly available eugenol-free material. It is mainly composed of zinc oxide, plaster of paris, and vinyl acetate. It provides good marginal seal when used to restore endodontic access preparations.^6^ However, there is paucity of researched information about the efficacy and properties of GC Caviton as a temporary restorative material. Hence, the aim of this *in vitro* study was to assess and compare the sealing properties, WS, and SL of IRM, Cavit G, and GC Caviton, which were used as temporary filling materials in coronal access openings.

## RESEARCH HYPOTHESIS

There will be difference in sealing properties, WS, and SL of IRM, Cavit G, and GC Caviton.

## MATERIALS AND METHODS

The study setting was the outpatient clinics of Department of Pedodontics and Preventive Dentistry at Bapuji Dental College and Hospital, Davangere, Karnataka, India. The study design was a double-blind randomized controlled trial, *in vitro* study.

Thirty-six extracted noncarious human premolars were selected for this study. All teeth were examined at 10c magnification, and those with microcracks were excluded. Ethical clearance was obtained by institutional review board (Ref no. BDC/Exam/393/2013-2014). Teeth were stored in 0.2% thymol solution for 7 days and then they were stored in saline.

Teeth were divided into three experimental groups:

- *Group I:* Intermediate restorative material
- *Group II:* Cavit G (Control)
- *Group III:* GC Caviton

This study was carried out in two parts.

### Part I: Evaluation of Sealing Ability

Standard endodontic access cavities of approximately 4 × 4 mm wide measured by periodontal probe were prepared through the occlusal surface. After preparing access, the pulp tissue was removed and the teeth were irrigated with 5.25% sodium hypochlorite, obturated with gutta-percha. To standardize the cavity depth, a periodon-tal probe was used to assure that it could accommodate 4 mm thickness of temporary material.^6^

Teeth were restored with experimental material including IRM (Dentsply Caulk, USA), Cavit G (3M ESPE, Germany), and GC Caviton (GC Corporation, Tokyo, Japan). The root apices were sealed with self-cured epoxy resin, and all the teeth were covered with two layers of nail varnish, leaving the restoration and 1 mm area surrounding them. The specimens were stored in saline for 7 days at 37°C. The specimens were thermocycled for 500 cycles between 5 ± 5 and 55 ± 5°C with 30 seconds dwell time and 3 seconds interval time. All the specimens were placed in 2% methylene blue dye for 24 hours, at room temperature. After washing, the specimens were sectioned into two parts along their longitudinal axis in a mesiodistal direction with a diamond disc. All specimens were viewed and photographed using a stereomicroscope (Leica, Germany) with 40i magnification. The greatest depth of dye penetration along the wall of the access cavity and the root of each hemisection was scored using scoring method.^7^

The scoring was done as follows:

- No visible dye penetration at the tooth/filling interface.
- Dye penetration limited to dentin-enamel junction.
- Dye penetration up to half of the pulp chamber.
- Dye penetration over half of the pulp chamber.^7^

### Part II: Evaluation of Water Absorption and Solubility

Disc-shaped 12 specimens for each group of 6 mm in diameter and 2 mm in height (h) were prepared for each material.

All specimens were stored in desiccator (Tempo Corporation, India) at 37°C, weighed daily to verify mass stabilization (dry mass, m_1_), and represented by mass variations lower than 0.1 mg in 24 hours interval. Thereafter, the specimens were stored in distilled water at 37°C for 7 days to obtain the mass after saturation with water (m_2_). The specimens were placed in the desiccator again at 37°C, and reweighed again until a constant dry mass is obtained (m_3_). Weighing was performed using analytical balance with 0.1 mg accuracy (Contech, India).

Water absorption and SL were determined by using formulas. Water absorption, given in μgmm^>3^, was calculated by using following formula:

WS = m_3_ - m_2_ / V

Solubility given in μg mm^-3^ was calculated by using the following formula:

SL = m_1_ - m_3_ / V

The volume (V) of each specimen was calculated based on the following equation, V = πR^2^h, where R is the specimen radius, h is the height of the specimen, ω is the constant.^7^

### Statistical Analysis

- For sealing ability, as data were nonparametric (ordinal), Kruskal-Wallis analysis of variance (ANOVA) was used for multiple group comparisons followed by Mann-Whitney U test.
- For WS and SL, one-way ANOVA analysis was used for intergroup comparison followed by *post hoc* Tukey’s test for group-wise comparison.
- Statistical analysis was done using Statistical Package for the Social Sciences (version 22).

## RESULTS

### Sealing Ability

**Graph 1:**
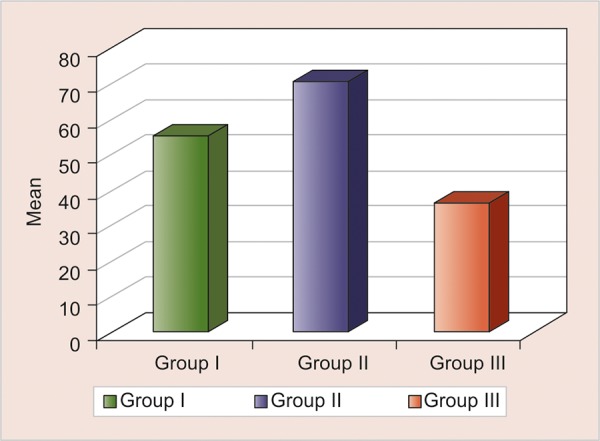
Intergroup comparison of sealing ability of three different groups

Least microleakage was shown in GC Caviton (36.78) followed by group IRM (55.78), and highest amount of microleakage was shown by Cavit G (70.94). The Kruskal-Wallis ANOVA revealed statistically significant differences between groups (p < 0.001, highly significant [HS]) (Table 1 and Graph 1) and also intergroup analysis using Mann-Whitney test revealed significant differences in all the groups (p < 0.005) (Table 2).

**Table 1:** **Table 1:** Comparison of sealing ability of three different groups using Kruskal-Wallis ANOVA test

*Groups*		*Samples (n)*		*Mean rank*		*Kruskal-Wallis* *ANOVA value*		*p-value*	
I		36		55.78		28.596		0.001*	
II		36		70.94					
III		36		36.78					

p < 0.001; *highly significant

**Table 2:** **Table 2:** *Post hoc* test comparison of sealing ability between the groups

*Groups*		*Mann-Whitney* *value*		*p-value* *significance*	
Group I *vs* group II		436		0.005*	
Group I *vs* group III		265		0.001**	
Group II *vs* group III		390		0.001**	

p < 0.005; *significant; p < 0.001; **highly significant

**Table 3:** **Table 3:** Comparison of water absorption of three different groups using one-way ANOVA test

*Groups*		*Samples (n)*		*Mean ± SD*		*ANOVA* *F-value*		*p-value*	
I		12		346.31 ± 196.65		14.826		0.001*	
II		12		565.60 ± 159.62					
III		12		235.83 ± 65.27					

*Highly significant; SD: Standard deviation

**Graph 2:**
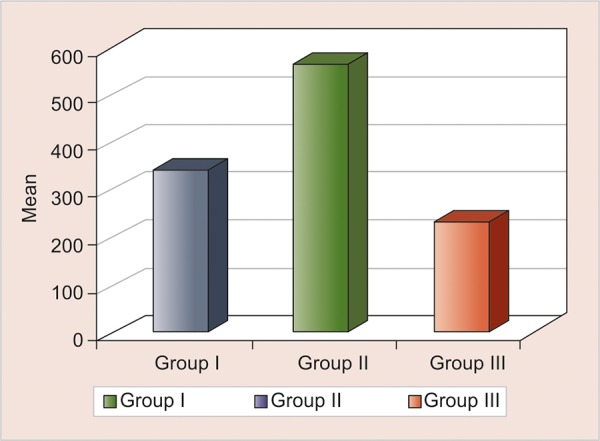
Intergroup comparison of water absorption of three different groups

**Table 4:** **Table 4:** *Post hoc* test comparison of water absorption between the experimental groups

*Groups*		*Mean difference* *(MD)*		*Standard* *error (SE)*		*p-value*	
Group I *vs* group II		219.291		61.65		0.003*	
Group I *vs* group III		110.483		61.65		0.188	
Group II *vs* group III		329.77		61.65		0.001**	

p < 0.005; *significant; **highly significant.

### Water Absorption

Least WS was shown in GC Caviton (235.83 ± 65.27) followed by IRM (346.31 ± 196.65), and highest amount of WS was shown by Cavit G (565.60 ± 159). Intergroup comparison by one-way ANOVA showed statistically significant differences between groups (p < 0.001, HS) (Table 3 and Graph 2), and also *post hoc* Tukey’s test for group-wise comparison revealed significant differences in all the groups (p < 0.005) (Table 4).

### Solubility

**Graph 3:**
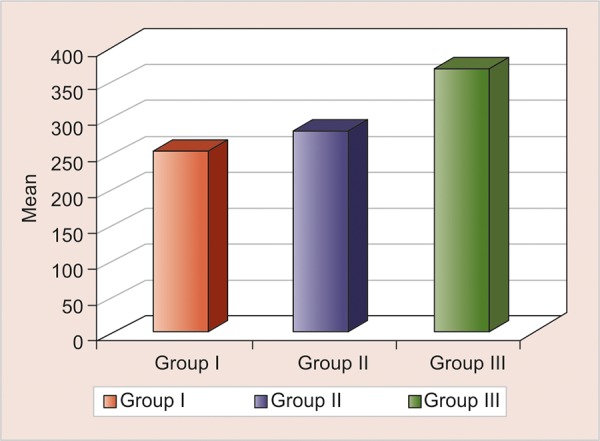
Intergroup comparison of SL of three different groups

The IRM showed least SL (256.0 ± 129.87) followed by Cavit G (285.0 ± 110.9) and GC Caviton (371.11 ± 135.54), and the p value was statistically nonsignificant (p > 0.005, NS) (Table 5 and Graph 3).

**Table 5:** **Table 5:** Comparison of SL of three different groups using oneway ANOVA test

*Groups*		*Samples (n)*		*Mean (±) SD*		*ANOVA* *F-value*		*p-value*	
I		12		256.0 ± 129.87		2.538		0.094	
II		12		285.33 ± 110.96					
III		12		371.11 ± 146.56					

p > 0.005; nonsignificant; SD: Standard deviation

## DISCUSSION

Methodological aspects of the test used in the study, namely methylene blue dye as leakage tracer, the thermocycling protocol, and the assessment of dye penetration through sections of the specimen, have been reported as the most frequent choices in marginal sealing evaluations.^8^ Only teeth free of caries, developmental defects, and enamel fractures and microfractures were included in the present study, as previous studies have revealed that any preexisting alteration of surface morphology of the tooth directly can influence the dye leakage.^9^ All access cavities were standardized and obturated, access cavities were filled with a thickness of 4 mm of material in accordance with the recommendation of Webber et al^10^ who found that a minimum depth of 3.5 mm of Cavit was required to prevent the total leakage of dye molecules. To ensure uniformity in the root canal length, the crown of the teeth could have been cut according to Magura et al,^11^ but the occlusal reduction exposes a significant number of the dentin tubules and leads to increased amount of microleakage; with the evidence of this study, the standardization of root canal length was obviated in the present study. Thus, only access cavity restoration thickness was standardized.^12^

In the present study, the specimens were thermo-cycled for 500 cycles between 5 ± 5 and 55 ± 5°C with 30 seconds dwell time and 3 seconds interval times; these variables seem to be tolerated by the oral tissues and are suitable for clinical conditions to simulate the intraoral temperature.^13^ Temperature changes in the oral cavity have been shown to adversely affect the marginal seal of dental material because the linear coefficients of thermal expansion of materials and dentin are different, perhaps representing the leading etiology of leakage.^14^ Methylene blue dye was used as a tracer for microleakage assessment because it is a more sensitive indicator of leakage and has a small molecular size, similar to that of the molecules of nutrients for microorganisms. Depth of dye penetration varied according to how much air was entrapped in the canal and it can easily penetrate by simple diffusion; it also has negligible influence on sealer of the root canal obturation. Moreover, it is not absorbed by the hydroxy-apatite crystal of dentin and it is frequently used for microleakage studies.^15^

In the present study, GC Caviton showed significantly better results compared with IRM and Cavit G, and this can be explained by the hygroscopic property of the material. GC Caviton is a ready-made cement mainly composed of zinc oxide, plaster of paris, and vinyl acetate. The good sealing ability of GC Caviton has been reported in the study done by Kim et al^16^ and Cruz et al,^6^ which is in agreement with the present study. Apart from this being in a premixed application, this also may reduce the inconsistence related to chair-side manipulation and adjusted in the access cavity. These good manipulation properties are considered as being supplementary factors for good coronal seal ability.^6^ In our study, the specimens were placed in saline immediately after cavity sealing in an attempt to mimic the actual clinical situation, which led to immediate hygroscopic expansion of the sealing material. The better sealing property of GC Caviton was achieved due to the expansion, which occurred by WS during cement setting, which caused the material to adhere closely to the cavity walls. In this study, it was found that IRM has poor sealing properties compared with GC Caviton. This is inconsistent with the finding reported by Magura et al,^11^ which might be attributed to lack of homogeneity and voids in IRM. In this study, high penetration of dye was found with Cavit G compared with GC Caviton, which is in agreement with the *in vitro* study reported by Kim et al.^16^ This can be explained by increased WS due to hydrophilic nature of material. These findings are in agreement with some of the reports published by Keller et al and Blaney et al.^17,18^

**Fig. 1:**
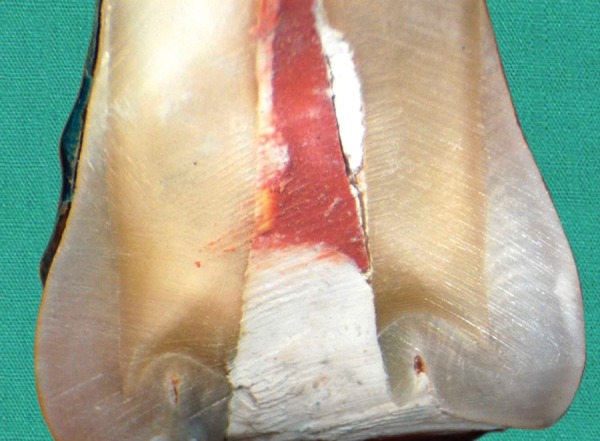
Microleakage score-0 seen in GC Caviton specimen No visible dye penetration at the tooth/filling interface

**Fig. 2:**
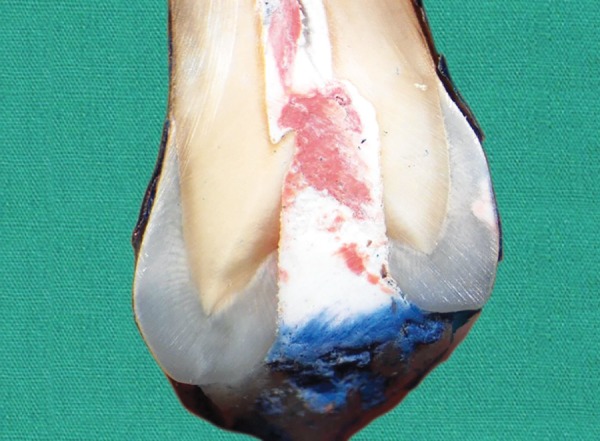
Microleakage score-1 seen in IRM specimen. Dye penetration limited to dentine- enamel junction

In the present study, Cavit G showed the highest amount of WS followed by IRM, and the least WS was reported by the GC Caviton. Water uptake is a key factor in the setting mechanism of Cavit G. The expansion caused by the water diffusion allows the swelling of components from the spaces occupied by water, explaining the high WS observed for this material in the present study, which is in agreement with the study performed by Cruz et al^6^ and Noguera and McDonald.^19^ Scanning electron observations by Tuna and Keyf^20^ reported that zinc oxide eugenol-free cement surfaces showed highest numbers of pores because of increased WS, which is consistent with our study. Although there was no significant difference in SL, the mean value of SL for IRM was low when compared with other two materials. Thus, IRM showed marginally less SL, which is in agreement with the study done by Poggio et al.^21^

**Fig. 3:**
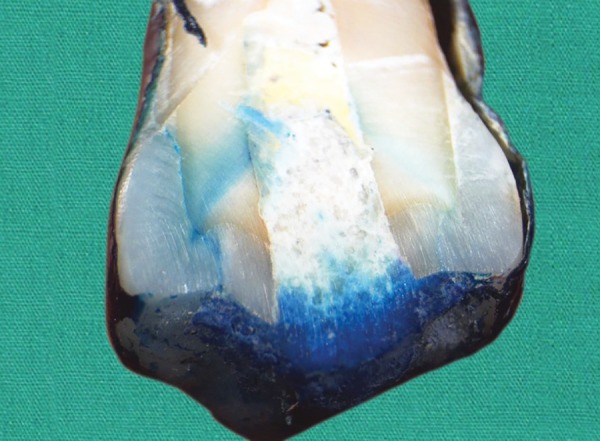
Microleakage score-2 seen in Cavit Gspecimen Dye penetration up to half of the pulp chamber

**Fig. 4:**
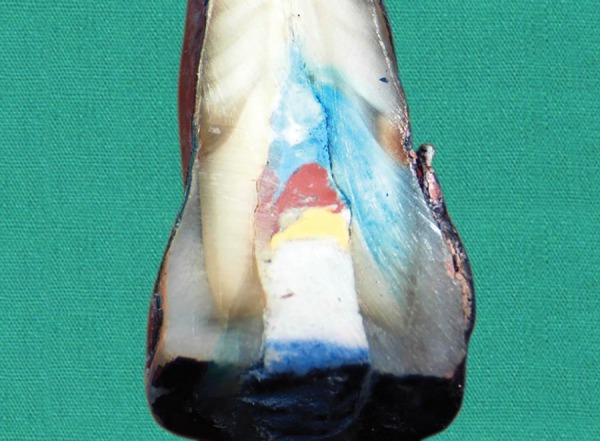
Microleakage score-3 seen in Cavit G specimen Dye penetration over half of the pulp chamber

## CONCLUSION

In the present study, GC Caviton has shown better sealing ability with least WS. Hence, within the limitations of the study it can be stated that GC Caviton proved to be an effective temporary restorative material during end-odontic treatment.

## References

[1] Naoum HJ, Chandler NP (2002). Temporization for endodontics.. Int Endod J.

[2] Sjogren U, Figdor D, Persson S, Sundqvist G (1997). Influence of infection at the time of root filling on the outcome of endodontic treatment of teeth with apical periodontitis.. Int Endod J.

[3] Shahi S, Samiei M, Rahimi S, Nezami H (2010). In vitro comparison of dye penetration through four temporary restorative materials.. Iran Endod J.

[4] Naseri M, Ahangari Z, Shahbazi Moghadam M, Mohammadian M (2012). Coronal sealing ability of three temporary filling materials.. Iran Endod J.

[5] Ciftcji A, Vardarli DA, Sonmez IS (2009). Coronal microleakage of four endodontic temporary restorative materials: an in vitro study.. Oral Surg Oral Med Oral Pathol Oral Radiol Endod.

[6] Cruz EV, Shigetani Y, Ishikawa K, Kota K, Iwaku M, Goodis HE (2002). A laboratory study of coronal microleakage using four temporary restorative materials.. Int Endod J.

[7] Pieper CM, Zanchi CH, Rodrigues-Junior SA, Moraes RR, Pontes LS, Bueno M (2009). Sealing ability, water sorption, solubility and toothbrushing abrasion resistance of temporary filling materials.. Int Endod J.

[8] Raskin A, D’Hoore W, Gonthier S, Degrange M, Dejou J (2001). Reliability of in vitro microleakage tests: a literature review.. J Adhes Dent.

[9] Ellwood RP, O’Mullane D (1996). The association between developmental enamel defects and caries in populations with and without fluoride in their drinking water.. J Public Health Dent.

[10] Webber RT, Del Rio CE, Brady JM, Segall RO (1978). Sealing quality of a temporary filling material.. Oral Surg Oral Med Oral Pathol.

[11] Magura ME, Kafrawy AH, Brown CE Jr, Newton CW (1991). Human saliva coronal leakage in obturated root canals: an in vitro study.. J Endod.

[12] Yun SM, Karanxha L, Kim H, Jung H, Park SX, Kyung (2012). Coronal microleakage of four temporary restorative materials in Class II-type endodontic access preparations.. Restor Dent Endod.

[13] Marosky JE, Patterson SS, Swartz M (1977). Marginal leakage of temporary sealing materials used between endodontic appointments and assessed by calcium 45 - an in vitro study.. J Endod.

[14] Anderson RW, Powell BJ, Pashley DH (1989). Microleakage of temporary restorations in complex endodontic access preparations.. J Endod.

[15] Zaia AA, Nakagawa R, De Quadros I, Gomes BP, Ferraz CC, Teixeira FB, Souza-Filho FJ (2002). An in vitro evaluation of four materials as barriers to coronal microleakage in root-filled teeth.. Int Endod J.

[16] Kim SY, Ahn JS, Yi YA, Lee Y, Hwang JY, Seo DG (2015). Quantitative microleakage analysis of endodontics temporary filling materials using a glucose penetration model.. Acta Odontol Scand.

[17] Keller DL, Peters DD, Setterstrom J, Bernier WE (1981). Microle-akage of softened temporary restorations as determined by microorganism penetration.. J Endod.

[18] Blaney TD, Peters DD, Setterstrom J, Bernier WE (1981). Marginal sealing quality of IRM and Cavit as assessed by microbiol penetration.. J Endod.

[19] Noguera AP, McDonald NJ (1990). Comparative in vitro coronal microleakage study of new endodontic restorative materials.. J Endod.

[20] Tuna SH, Keyf F (2006). Water sorption and solubility of provisional and permanent luting cement.. Hacettepe Dishekimligi Fakul- tesi Dergisi.

[21] Poggio C, Lombardini M, Alessandro C, Simonetta R (2007). Solubility of root-end-filling materials: a comparative study.. J Endod.

